# Nanocurcumin attenuates brain injury in rats with desert dry-heat-induced exertional heat stroke by suppressing TLR4/MyD88/NF-κB signaling

**DOI:** 10.3892/mmr.2026.13953

**Published:** 2026-07-02

**Authors:** Lisong Su, Jinquan Qu, Jiajia Li, Wenhui Shi, Laiyang Song, Feixing Liang, Jiangwei Liu

**Affiliations:** Desert Medicine Laboratory, General Hospital of Xinjiang Military Command, Ürümqi, Xinjiang Uygur Autonomous Region 830000, P.R. China

**Keywords:** exertional heat stroke, desert dry-heat, nanocurcumin, brain injury, neuroinflammation, TLR4/MyD88/NF-κB signaling

## Abstract

Exertional heat stroke (EHS) is a life-threatening condition characterized by hyperthermia, systemic inflammation and central nervous system injury, particularly in desert dry-heat environments. Excessive activation of inflammatory signaling pathways, notably the Toll-like receptor (TLR)4/myeloid differentiation factor 88 (MyD88)/NF-κB axis, critically contributes to brain damage and neuroendocrine dysfunction in EHS. Curcumin exhibits anti-inflammatory and neuroprotective effects; however, poor bioavailability limits its clinical application. Notably, nanocrystal formulations may improve the therapeutic efficacy of curcumin. In the present study, network pharmacology and molecular docking were employed to identify the potential therapeutic targets of curcumin in EHS. A rat model of desert dry-heat-induced EHS was established and nanocurcumin was administered intravenously following heat exposure. Histopathological examination, ELISA analyses of neuroendocrine hormones and inflammatory cytokines, serum biochemical assays and western blotting were subsequently performed. These evaluations assessed brain injury, hypothalamic-pituitary-adrenal and hypothalamic-pituitary-thyroid axes functions, systemic inflammation, peripheral organ injury indicators and activation of the TLR4/MyD88/NF-κB signaling pathway. Network analysis revealed 138 overlapping target genes between curcumin and EHS, identifying AKT1, TNF, EGFR, BCL2, STAT3, SRC and NFKB1 as key hub genes. Kyoto Encyclopedia of Genes and Genomes pathway analysis highlighted enrichment of the ‘Toll-like receptor signaling pathway’ and ‘NF-κB signaling pathway’. Molecular docking indicated favorable binding affinities of curcumin to essential inflammatory proteins, including TLR4, MyD88 and NFKB1. *In vivo* experiments demonstrated that nanocurcumin reduced neuronal injury in the cerebral cortex and hypothalamus of rats. Furthermore, nanocurcumin significantly decreased serum concentrations of corticotropin-releasing hormone, corticosterone, thyrotropin-releasing hormone and thyroid-stimulating hormone, and restored adrenocorticotropic hormone, total triiodothyronine and free triiodothyronine levels. Nanocurcumin also lowered serum TNF-α, IL-6 and IL-1β levels, and improved biochemical markers of liver, kidney and tissue injury (alanine aminotransferase, aspartate aminotransferase, blood urea nitrogen, creatine kinase and lactate dehydrogenase). Within the 4-h observation period, medium and high doses of nanocurcumin did not worsen biochemical markers compared with those in the EHS or saline groups. Additionally, nanocurcumin administration dose-dependently inhibited TLR4, MyD88 and NF-κB protein expression in brain tissues. In conclusion, nanocurcumin may alleviate brain injury, neuroendocrine dysfunction and systemic inflammation associated with desert dry-heat-induced EHS, and may improve biochemical indicators of peripheral organ damage; these effects likely involve suppression of the TLR4/MyD88/NF-κB signaling pathway. These findings support the use of nanocurcumin as a promising adjunctive therapy for managing EHS.

## Introduction

Exertional heat stroke (EHS) is a life-threatening medical emergency induced by strenuous physical activity under high-temperature conditions (1). EHS is characterized by a rapid increase in core body temperature, typically exceeding 40.5°C, accompanied by central nervous system (CNS) dysfunction, including delirium, seizures or coma (2). In desert dry-heat environments, intense thermal radiation, low humidity and marked diurnal temperature variations can further intensify thermal stress, accelerating the progression of EHS and exacerbating brain injury (3). The pathogenesis of EHS involves a complex interplay among systemic inflammatory response syndrome, endothelial dysfunction, coagulation abnormalities and multi-organ failure. Among these mechanisms, excessive activation of inflammatory signaling pathways serves a central role. Specifically, heat stress activates the Toll-like receptor (TLR)4/myeloid differentiation factor 88 (MyD88) signaling pathway, leading to NF-κB activation, subsequent release of pro-inflammatory mediators, such as TNF-α, IL-6 and IL-1β, which further contribute to neuroinflammation, disruption of the blood-brain barrier and neuronal injury (4,5). Therefore, targeting inflammatory signaling pathways, such as the TLR4/NF-κB axis, may represent a promising therapeutic approach for EHS-induced brain injury.

Curcumin (6), a polyphenolic compound derived from *Curcuma longa*, exhibits potent anti-inflammatory, antioxidant and neuroprotective properties. Previous studies have demonstrated that curcumin inhibits activation of the TLR4/NF-κB signaling pathway, suppresses pro-inflammatory cytokine production, and reduces oxidative stress in various inflammatory and neurological disease models (7–9). These findings suggest that curcumin may have therapeutic potential in alleviating neuroinflammation and brain injury associated with EHS.

Notably, the clinical translation of curcumin is limited by its poor aqueous solubility, rapid metabolism and low systemic bioavailability (9). To overcome these limitations, nanotechnology-based formulations have been developed to enhance pharmacokinetic properties. Specifically, nanocurcumin in the form of nanocrystals markedly improves solubility, stability and tissue distribution, without altering the intrinsic chemical structure of curcumin (10,11). Intravenous nanocrystal formulations may thus enhance the therapeutic efficacy of curcumin in acute pathological conditions such as EHS.

While nanocrystal technology improves drug delivery efficiency, the pharmacodynamic targets remain determined by the molecular structure of curcumin. Therefore, the present study employed network pharmacology based on the molecular structure of curcumin to predict potential therapeutic targets in EHS. Subsequently, *in vivo* experiments utilized intravenous nanocrystal formulations to enhance the bioavailability and therapeutic effectiveness of curcumin (11).

Considering the multifactorial pathogenesis of EHS and the multitarget characteristics of curcumin, combining network pharmacology with experimental validation may provide a more comprehensive understanding of the therapeutic mechanisms of curcumin. Thus, the present study aimed to investigate the protective effects of intravenously administered nanocurcumin against desert dry-heat-induced EHS-related brain injury in rats, focusing specifically on modulation of the TLR4/MyD88/NF-κB signaling pathway and associated neuroendocrine dysfunction.

## Materials and methods

### Ethical considerations and animals

A total of 48 specific pathogen-free male Sprague-Dawley rats (age, 6–8 weeks; weight, 250–320 g) were obtained from the Experimental Animal Center of Xinjiang Medical University (Ürümqi, China). All experimental protocols adhered to international guidelines for the care and use of laboratory animals (12) and were approved by the Animal Experiment Ethics and Welfare Committee of General Hospital of Xinjiang Military Command (ethics no. DWLL2023021802; Ürümqi, China).

The rats were housed in a small animal facility at the Desert Medicine Laboratory, General Hospital of Xinjiang Military Command, under controlled conditions (temperature: 22±2°C; relative humidity, 40±5%; 12 h light/12 h dark cycle). Before experimentation, rats underwent a 7-day adaptive period with unrestricted access to food and water.

### Model establishment and grouping

Experiments were conducted in the Simulated Climate Cabin for Special Environment of Northwest China (Ürümqi, China). The environmental parameters for the experimental groups were set at 41±1°C temperature and 15±5% relative humidity, whereas the control group was maintained under standard conditions (22±2°C, 40±5% relative humidity). Before modeling, all rats underwent 7 days of incremental-load adaptive training on a horizontal treadmill. The training schedule was as follows: Treadmill speed was set at 10 m/min on day 1, at 12 m/min on day 2, was increased by 1 m/min daily between days 3 and 5 (up to 14 m/min), and was stabilized at 15 m/min for days 6 and 7. Each training session lasted 15 min. To encourage continuous exercise, a constant current stimulation electrode (1 mA) was placed at the end of the treadmill for negative reinforcement (13). After adaptive training, the rats were randomly allocated into six groups (n=8/group) using stratified randomization: Control, EHS, physiological saline, and low-, medium- and high-dose nanocurcumin groups. Based on reported safe intravenous curcumin dosages in humans (120 mg) (14,15), rat doses of 9.75, 19.5 and 29.25 mg/kg were calculated using standard body surface area normalization.

During EHS modeling undertaken by all rats, the rats exercised at 15 m/min in a desert environment (41±1°C temperature, 15±5% relative humidity). Rectal temperatures were measured and recorded every 5 min. The endpoint criteria for successful modeling included core temperatures of ≥42.5°C in three consecutive measurements at 5-min intervals, accompanied by exercise incapacity (loss of righting reflex). Upon reaching these criteria, the rats were immediately removed from the heat chamber.

After successful establishment of the model, nanocurcumin was administered intravenously in a nanocrystal formulation (particle size, ~70 nm; concentration, 30 mg/ml), provided by the Institute of Pharmacology and Toxicology, Academy of Military Medical Sciences (Beijing, China). The formulation was diluted in sterile normal saline to achieve the desired concentrations. All injections were administered via the tail vein immediately after EHS induction (post-treatment intervention). The saline group received an equivalent volume of sterile normal saline. The control group received no treatment and was kept under normal conditions without EHS induction or drug administration. Rats were euthanized 4 h after nanocurcumin administration for sample collection.

### Blood sample collection and examination

After the observation period, the rats were anesthetized intraperitoneally with pentobarbital sodium (30 mg/kg) and secured on an operating table. Blood samples (3–5 ml per rat) were collected from the abdominal aorta under deep anesthesia. After blood collection, the rats were humanely euthanized by exsanguination under continued anesthesia. Death was confirmed by the absence of heartbeat, respiratory movements, corneal reflex and response to paw pinch prior to tissue collection. All procedures adhered strictly to institutional guidelines for laboratory animal care and use, and humane endpoints were predefined. Humane endpoints included severe neurological dysfunction (for example, loss of righting reflex, seizure activity, or unresponsiveness), marked impairment of motor function (such as inability to stand or maintain posture), signs of severe physiological distress (for example, abnormal respiration patterns and profound weakness), and any condition judged by veterinary staff to indicate moribund state or irreversible distress. No animals died spontaneously or before the planned experimental endpoint. Blood samples were collected from the abdominal aorta into vacuum collection tubes containing appropriate anticoagulants. Brain tissues were collected on ice, immediately transferred to cryogenic tubes and placed in fixative solutions.

Serum samples were allowed to clot at room temperature for 30 min, after which they were centrifuged at 1,000 × g, at 4°C for 15 min, and the supernatants were collected for biochemical and ELISA analyses. Aliquots of serum samples were stored at −80°C until further analysis.

### Serological analysis

Serum levels of alanine aminotransferase (ALT), aspartate aminotransferase (AST), creatinine (Cr), blood urea nitrogen (BUN), creatine kinase (CK) and lactate dehydrogenase (LDH) were measured using an automated biochemical analyzer (Shenzhen Mindray Bio-Medical Electronics Co., Ltd.). Concentrations of IL-6, TNF-α, IL-1β, thyrotropin-releasing hormone (TRH), thyroid-stimulating hormone (TSH), corticotropin-releasing hormone (CRH), adrenocorticotropic hormone (ACTH), total triiodothyronine (T3), total thyroxine (T4), free triiodothyronine (FT3), free thyroxine (FT4) and corticosterone (CORT) were determined using rat ELISA kits following the manufacturer's protocols. IL-6 (cat. no. F3066-A), TNF-α (cat. no. F3056-A), IL-1β (cat. no. F2923-A), TRH (cat. no. F3393-A), ACTH (cat. no. F3440-A), T3 (cat. no. F0487-OA), FT3 (cat. no. F0004-ZA), T4 (cat. no. F0107-ZA), FT4 (cat. no. F0010-ZA) and CORT (cat. no. F0096-OA) kits were purchased from Fankewei Biotechnology Co., Ltd. TSH was measured using a kit from Beijing Bioss Biotechnology Co., Ltd. (cat. no. BSKR62969), and CRH was measured using a kit from Wuhan Saipei Biotechnology Co., Ltd. (cat. no. SP13191). Each sample was tested in duplicate. Absorbance was measured at 450 nm using a microplate reader, and concentrations were calculated from standard curves.

### Histological examination

Brain tissues were fixed in 10% neutral buffered formalin at room temperature (20–25°C) for 7 days, followed by dehydration and paraffin embedding according to standard protocols. Paraffin-embedded tissues were sectioned at 4-µm thickness. For H&E staining, paraffin sections were deparaffinized in xylene and rehydrated through graded ethanol. Sections were stained with hematoxylin solution for 3–5 min at room temperature, followed by eosin staining for 1–2 min at room temperature using a Servicebio H&E staining kit (Wuhan Servicebio Technology Co., Ltd.), according to the manufacturer's instructions. For Nissl staining, sections were incubated in cresyl violet staining solution (Wuhan Servicebio Technology Co., Ltd.) for 2–5 min at room temperature, followed by differentiation in 0.1% acetic acid and dehydration, according to the manufacturer's instructions. After staining, sections were dehydrated, cleared and mounted with neutral resin. Images were acquired using a light microscope.

### Network pharmacology analysis

The 3D structure of curcumin was retrieved from the PubChem database (https://pubchem.ncbi.nlm.nih.gov/). Potential targets of curcumin were predicted using SwissTargetPrediction (http://www.swisstargetprediction.ch), similarity ensemble approach (SEA; http://sea.bkslab.org) and PharmMapper (16). For SEA analysis, target screening was limited to ‘*Homo sapiens*’ with a Max-Tc score >0.8. For PharmMapper, targets with a Fit Score ≥3.0 and P<0.05 were selected. Results from these databases were merged, duplicates removed and a comprehensive curcumin-target dataset was established. EHS-related targets were collected from GeneCards (https://www.genecards.org), OMIM (https://www.omim.org) and National Center for Biotechnology Information (NCBI) Gene (https://www.ncbi.nlm.nih.gov/gene) using the key word ‘exertional heat stroke’. After duplicates were removed, an EHS-target dataset was created. Potential therapeutic targets of curcumin for EHS were identified using the Jvenn online tool (https://www.bioinformatics.com.cn/static/others/jvenn/) by intersecting the curcumin-target and EHS-target datasets.

Protein-protein interaction (PPI) analysis was conducted using the STRING database (version 11.5; http://string-db.org), restricting species to ‘*Homo sapiens*’ with a confidence score ≥0.4. The resulting PPI network was imported into Cytoscape software (version 3.10.4; http://cytoscape.org/) (17) for visualization and topological analysis. Hub genes were identified using the CytoHubba plugin based on degree ranking, reflecting the number of direct interactions with other nodes. Significant functional modules within the network were further classified using the MCODE plugin in Cytoscape with the parameters set as follows: Degree Cutoff, 2; Node Score Cutoff, 0.2; K-Core, 2; and Max Depth, 100. Modules demonstrating high connectivity and biological relevance were selected for subsequent analyses.

Gene Ontology (GO) and Kyoto Encyclopedia of Genes and Genomes (KEGG) enrichment analyses were conducted using the clusterProfiler package in R (version 4.5.2; http://www.r-project.org/) (18). The Benjamini-Hochberg method was used to correct for multiple testing, and adjusted P-values (P.adjust <0.05) were considered statistically significant.

### Molecular docking

Three-dimensional structures of hub proteins were retrieved from the Protein Data Bank (https://www.rcsb.org/). Protein structures were processed using PyMOL (version 2.3.0; http://pymol.org/) by removing water molecules and ligands, hydrogen atoms were added using AutoDock Tools (version 1.5.7; http://autodock.scripps.edu/) and structures were saved in pdbqt format. The 3D structure of curcumin was downloaded from PubChem and optimized using the MMFF94 force field in OpenBabel (version 3.1.1; http://openbabel.org/) to obtain its lowest-energy conformation. Molecular docking was performed using AutoDock Vina (version 1.2.5; http://vina.scripps.edu/) with an exhaustiveness setting of 25 to ensure docking accuracy. Binding affinities were evaluated based on calculated binding free energies (kcal/mol), where lower values indicate stronger binding.

### Western blotting

Approximately 50 mg brain tissue was ground in liquid nitrogen and transferred into a 1.5-ml centrifuge tube. The tissue samples were then lysed in 300 µl RIPA buffer (Wuhan Servicebio Technology Co., Ltd.) containing 1 mM PMSF, 1X phosphatase inhibitor cocktail and 1X protease inhibitor cocktail. After incubation on ice for 30 min, the lysates were centrifuged at 12,000 × g for 20 min at 4°C, and supernatants were collected.

Protein concentrations were measured using a BCA protein assay kit (Wuhan Servicebio Technology Co., Ltd.) according to the manufacturer's instructions. Subsequently, equal amounts of protein (30 µg/lane) were separated by SDS-PAGE on 10% gels and transferred onto PVDF membranes. The membranes were blocked in 5% skimmed milk prepared in TBST (Tris-buffered saline containing 0.1% Tween-20) at room temperature for 1 h. The membranes were incubated overnight at 4°C with primary antibodies against TLR4 (cat. no. BS-1021R; 1:1,000; BIOSS), MyD88 (cat. no. BS-1047R; 1:1,000; BIOSS), NF-κB (cat. no. BS-0465R; 1:1,000; BIOSS) and β-actin (cat. no. 4970S; 1:2,000; Cell Signaling Technology, Inc.). After three washes in TBS-Tween (10 min each), the membranes were incubated with HRP-conjugated secondary antibodies (goat anti-rabbit IgG; cat. no. A0208; 1:10,000; Wuhan Servicebio Technology Co., Ltd.) for 1 h at room temperature. The protein bands were visualized using enhanced chemiluminescence (ECL) detection kit (Wuhan Servicebio Technology Co., Ltd.) reagent and captured using a gel imaging system. Band intensities were semi-quantified using ImageJ software (version 1.53t; National Institutes of Health) and normalized to β-actin.

### Statistical analysis

All statistical analyses were performed using SPSS 25.0 (IBM Corp.), GraphPad Prism 10 (Dotmatics) and R 4.5.2. Data normality was assessed using the Shapiro-Wilk test and homogeneity of variance was evaluated using Levene's test. Data are presented as the mean ± SD. Differences among groups were analyzed using one-way ANOVA, followed by Tukey's post hoc test for multiple comparisons. All tests were two-sided and P<0.05 was considered to indicate a statistically significant difference.

## Results

### Identification of potential therapeutic targets

**Figure 1.**
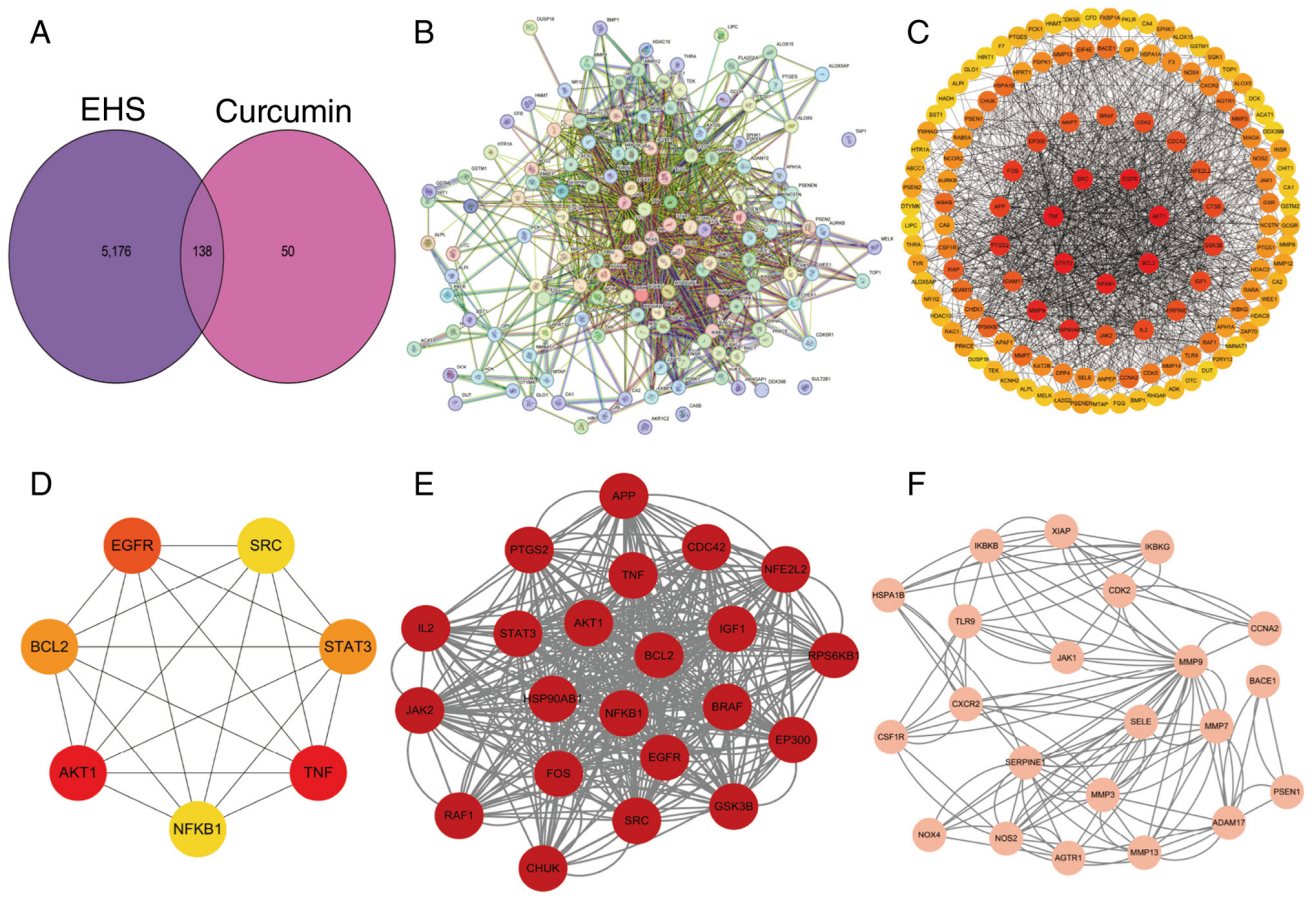
Identification and network pharmacological analysis of potential curcumin targets for EHS treatment. (A) Venn diagram illustrating overlapping targets between curcumin and EHS-related genes. (B) PPI network constructed from the overlapping targets. (C) Modular analysis highlighting core subnetworks within the PPI network. The inner core cluster (red nodes) indicates a densely connected primary target module, while the outer circle (orange-yellow nodes) represents closely associated peripheral modules. (D) Interaction network of hub genes. The top seven hub genes (EGFR, SRC, BCL2, STAT3, AKT1, TNF and NFKB1), identified using the CytoHubba plugin, and their direct interactions are shown. (E) Core functional module (cluster 1) identified by MCODE analysis. (F) Module related to extracellular matrix remodeling and inflammation (cluster 3) identified by MCODE analysis. EHS, exertional heat stroke; PPI, protein-protein interaction.

A total of 188 potential curcumin targets were identified from the SwissTargetPrediction and SEA databases. Additionally, 5,314 EHS-related target genes were retrieved from the GeneCards and NCBI databases. Venn analysis identified 138 overlapping targets, which were considered potential therapeutic targets of curcumin in EHS (Fig. 1A). These intersecting targets were imported into the STRING database to construct a PPI network (Fig. 1B). Topological analysis using Cytoscape 3.10.4 showed that the PPI network comprised 134 nodes and 2,392 edges (Fig. 1C). Based on node degree, the CytoHubba plugin was used to identify the top seven hub genes: AKT1, TNF, EGFR, BCL2, STAT3, SRC and NFKB1 (Fig. 1D). These hub genes are primarily involved in inflammatory regulation, cell survival, apoptosis and signal transduction (19–23), suggesting that curcumin may exert therapeutic effects against EHS through multiple key signaling targets.

To further characterize the PPI network and improve visualization of the results, MCODE analysis was performed to identify highly interconnected functional modules. Six significant clusters were identified (Table I). Among these, clusters 1 and 3 were selected for further analysis because of their high connectivity and biological relevance.

Cluster 1 had the highest MCODE score (19.143) and contained 22 nodes and 402 edges, indicating that it represented the core functional module. This cluster included several key genes, including BRAF, FOS, STAT3, TNF, SRC, EGFR, AKT1, JAK2, BCL2, CHUK and NFKB1 (Fig. 1E). These genes were mainly associated with inflammation-related signaling pathways, including the NF-κB, PI3K-AKT, MAPK and JAK-STAT pathways (24,25).

Cluster 3 contained 22 nodes and 126 edges. Representative genes in this cluster included MMP3, MMP7, MMP9, MMP13, ADAM17, NOS2, NOX4, TLR9, IKBKB and SERPINE1 (Fig. 1F). These genes were primarily associated with extracellular matrix remodeling, inflammatory responses and oxidative stress-related processes (26). The remaining clusters were not analyzed further because they exhibited lower connectivity or were less relevant to the primary biological processes investigated in the current study.

### GO and KEGG enrichment analyses

**Figure 2.**
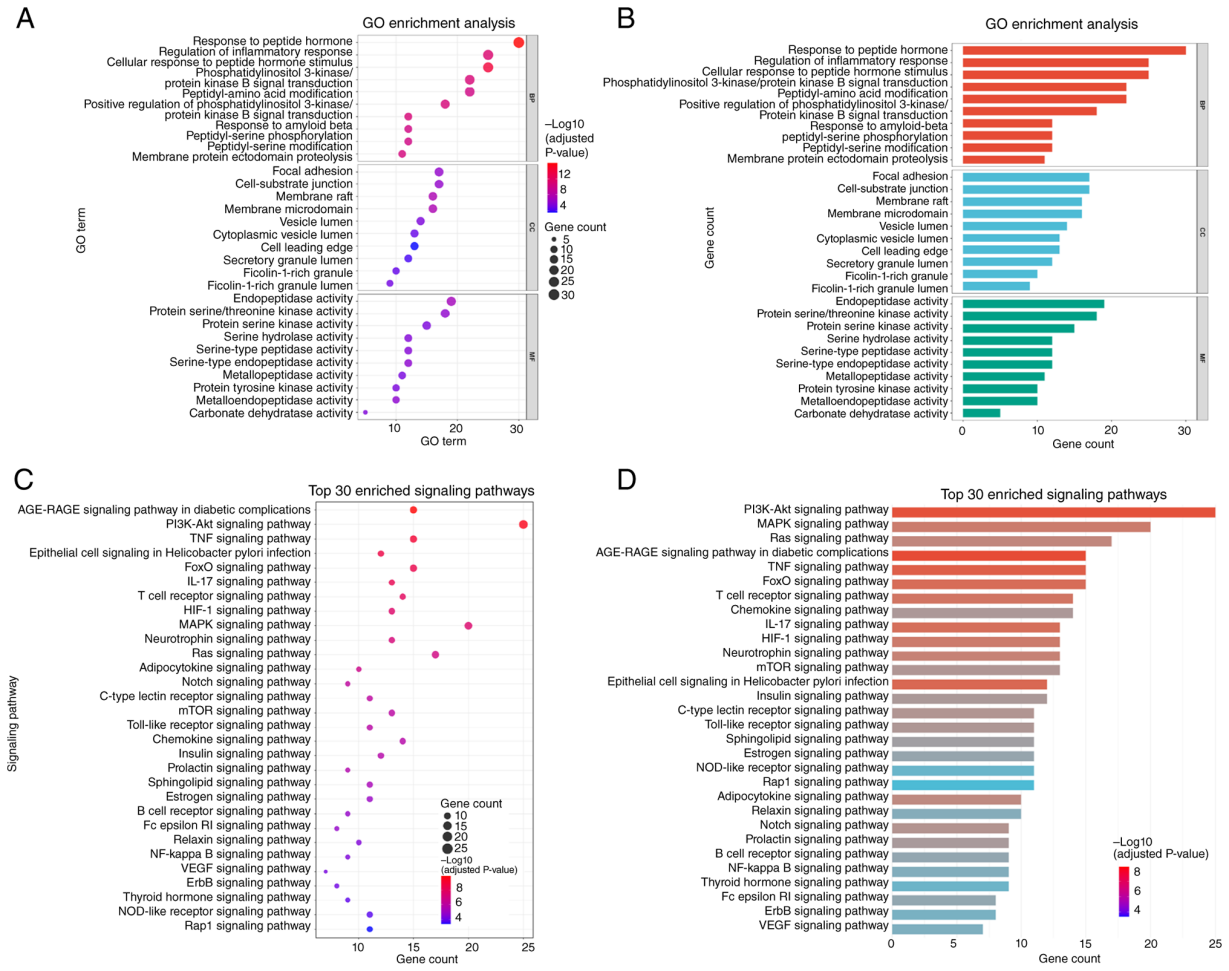
Gene function and pathway enrichment analyses of the potential therapeutic targets of curcumin in exertional heat stroke. (A) Bubble plot depicting GO enrichment of overlapping targets, displaying the top 10 significantly enriched terms across BP, CC and MF categories. Bubble size represents gene count and color intensity indicates enrichment significance. (B) Bar plot of GO enrichment results for overlapping targets. (C) KEGG pathway enrichment bubble plot, showing the top 30 significantly enriched pathways. Bubble size corresponds to gene count and color intensity indicates enrichment significance. (D) Bar plot illustrating KEGG pathway enrichment for overlapping targets. BP, biological processes; CC, cellular component; GO, Gene Ontology; KEGG, Kyoto Encyclopedia of Genes and Genomes; MF, molecular function.

The 138 overlapping targets between curcumin and EHS were subjected to GO and KEGG enrichment analyses. GO analysis (Fig. 2A and B) showed that these targets were predominantly enriched in biological processes related to ‘response to peptide hormone’, ‘regulation of inflammatory response’, ‘cellular response to peptide hormone stimulus’, ‘response to stress’, and ‘cellular response to stimulus’. In the cellular component category, significant enrichment was observed in ‘membrane raft’ and ‘membrane microdomain’, as well as ‘focal adhesion’ and ‘cell-substrate junction’, suggesting involvement in receptor-mediated signaling complex assembly. Molecular function analysis revealed significant enrichment in ‘signal transducer activity’ and ‘cytokine receptor binding’.

KEGG pathway analysis (Fig. 2C and D) demonstrated that the overlapping targets were significantly enriched in multiple signaling pathways, including ‘PI3K-Akt signaling pathway’, ‘MAPK signaling pathway’, ‘Ras signaling pathway’, ‘AGE-RAGE signaling pathway in diabetic complications’, ‘TNF signaling pathway’, ‘FoxO signaling pathway’, ‘T cell receptor signaling pathway’, ‘Chemokine signaling pathway’, ‘IL-17 signaling pathway’, ‘HIF-1 signaling pathway’, ‘mTOR signaling pathway’, ‘Insulin signaling pathway’, ‘NF-kappa B signaling pathway’, ‘Toll-like receptor signaling pathway’, ‘VEGF signaling pathway’, ‘Notch signaling pathway’, and ‘B cell receptor signaling pathway’. These pathways are mainly involved in inflammatory regulation, immune response and cell survival signaling. These pathways are known to serve central roles in the systemic inflammatory response induced by EHS (1,7,27,28). Given the pivotal role of the TLR4/MyD88/NF-κB axis in amplifying inflammatory cascades, this pathway was selected for subsequent experimental validation. Collectively, these enrichment analyses suggested that curcumin may protect against EHS-induced brain injury through modulation of key inflammatory signaling networks.

### Molecular docking

**Figure 3.**
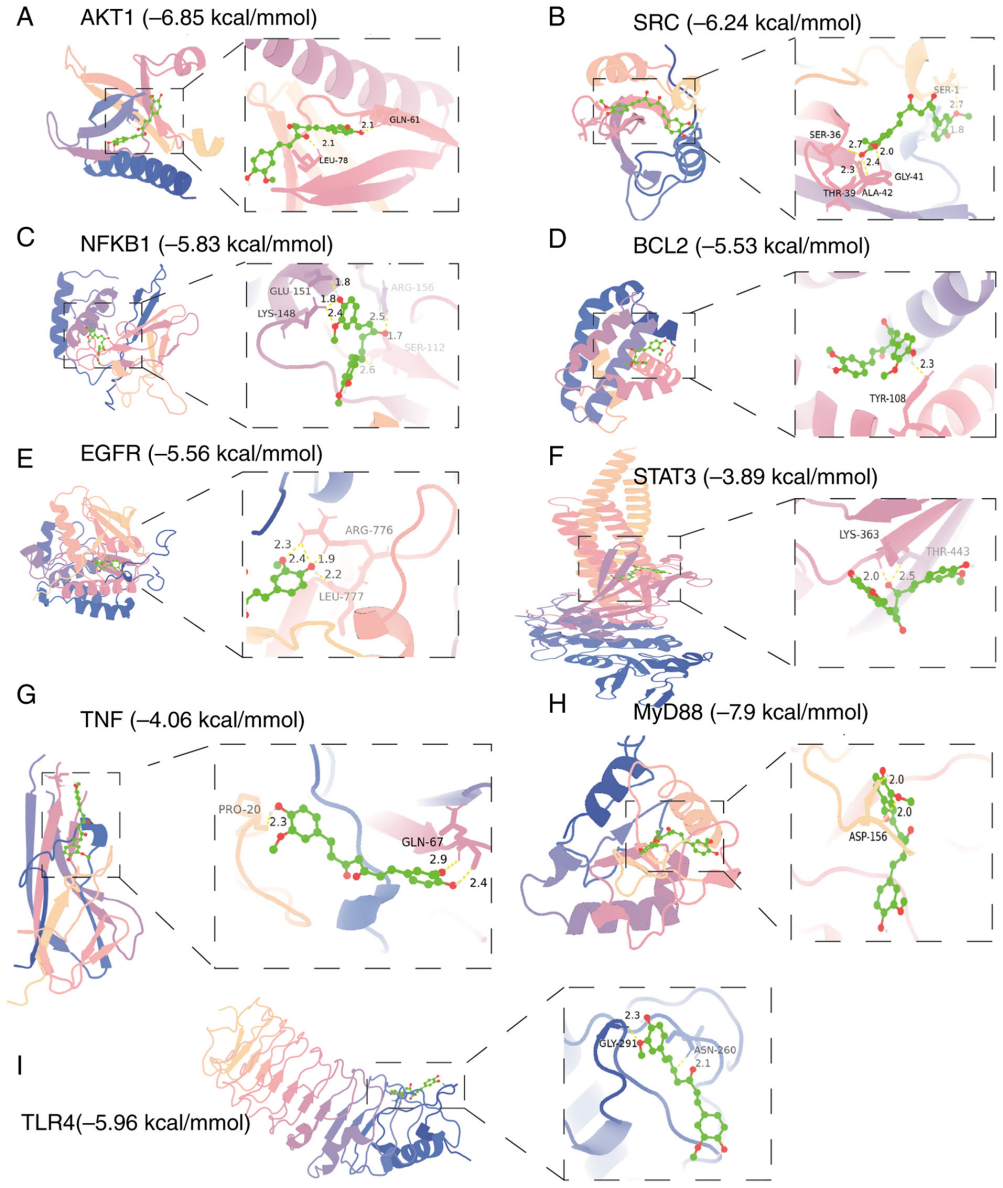
Molecular docking interactions between curcumin and key hub targets. Representative molecular docking images illustrating the binding interactions of curcumin with nine key target proteins: (A) AKT1, (B) SRC, (C) NFKB1, (D) BCL2, (E) EGFR, (F) STAT3, (G) TNF, (H) MyD88 and (I) TLR4. MyD88, myeloid differentiation factor 88; TLR4, Toll-like receptor 4.

To further investigate interactions between key target proteins and curcumin in the context of EHS treatment, molecular docking analysis was conducted. The docking results indicated that curcumin exhibited strong binding affinities with the active pockets of the identified target proteins. Interaction network analysis highlighted AKT1, SRC, NFKB1, BCL2, EGFR, STAT3 and TNF as highly interconnected nodes, potentially serving critical roles in the pathogenesis of EHS. Curcumin demonstrated favorable binding to these proteins, with calculated binding free energies of −6.85 kcal/mol (AKT1), −6.24 kcal/mol (SRC), −5.83 kcal/mol (NFKB1), −5.53 kcal/mol (BCL2), −5.56 kcal/mol (EGFR), −3.89 kcal/mol (STAT3) and −4.06 kcal/mol (TNF-α) (Fig. 3A-G). These data suggested that curcumin may modulate the activity of these target proteins by binding directly to their receptor sites, thereby potentially attenuating pathological processes associated with EHS. Additionally, based on KEGG pathway enrichment analysis highlighting the TLR and NF-κB signaling pathways, docking analyses were conducted for MyD88 and TLR4, two key upstream regulators of NF-κB signaling. Curcumin exhibited strong binding affinities with MyD88 (−7.9 kcal/mol) and TLR4 (−5.96 kcal/mol) (Fig. 3H and I). Collectively, these findings indicated that curcumin may exert therapeutic effects against EHS through interactions with these critical molecular targets, including key upstream regulators of the TLR4 signaling pathway.

### Nanocurcumin alleviates EHS-induced endocrine axis dysfunction

**Figure 4.**
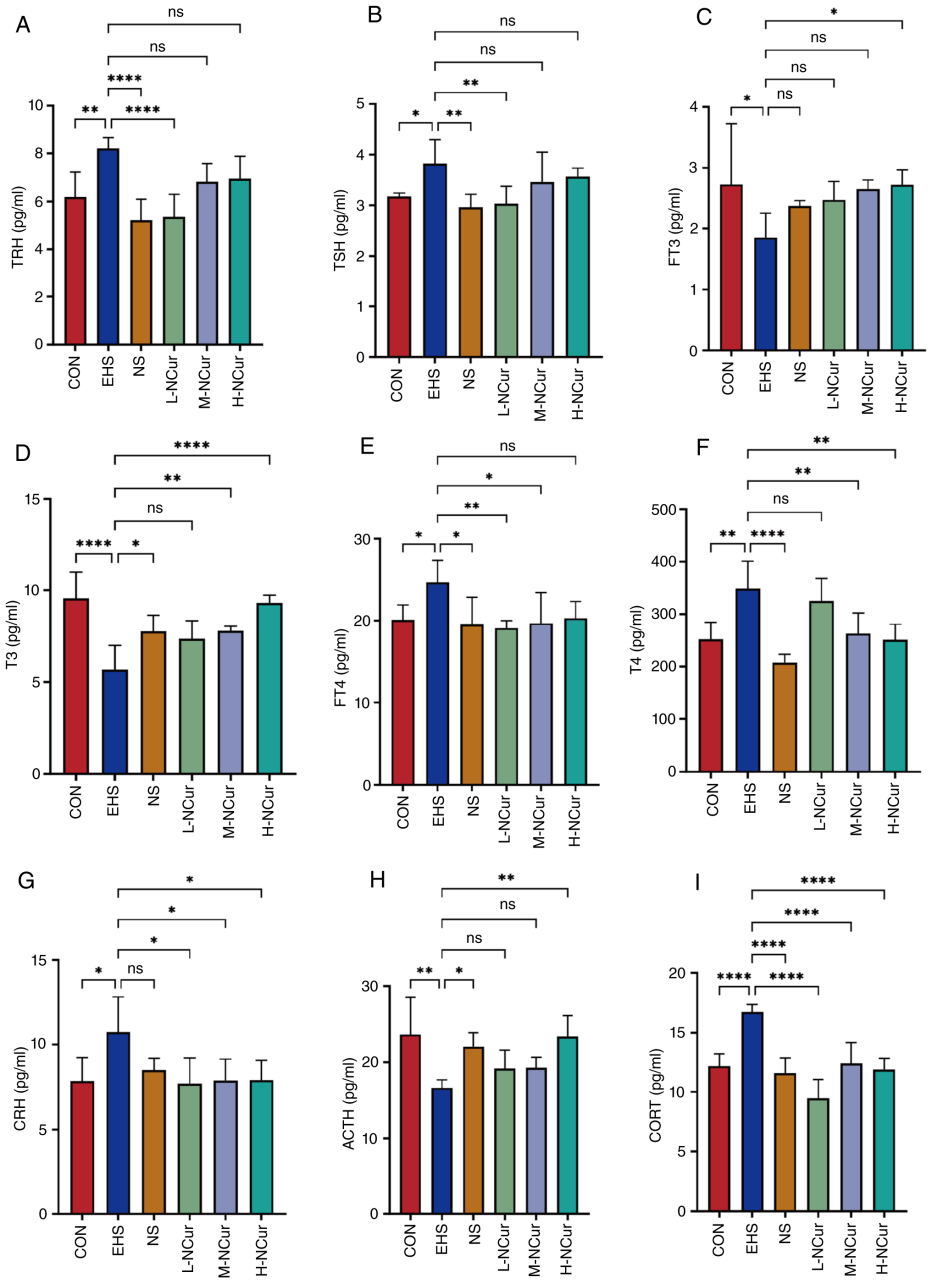
Effect of nanocurcumin on serum neuroendocrine hormone levels in rats with EHS. Serum concentrations of (A) TRH, (B) TSH, (C) FT3, (D) T3, (E) FT4, (F) T4, (G) CRH, (H) ACTH and (I) CORT across different experimental groups. Data are presented as the mean ± SD. Statistical analyses were conducted using one-way ANOVA with Tukey's post hoc test. *P<0.05, **P<0.01, ****P<0.0001; ns, not significant. ACTH, adrenocorticotropic hormone; CON, control; CORT, corticosterone; CRH, corticotropin-releasing hormone; EHS, exertional heat stroke; FT3, free triiodothyronine; FT4, free thyroxine; H-NCur, high-dose nanocurcumin; L-NCur, low-dose nanocurcumin; M-NCur, medium-dose nanocurcumin; NS, normal saline; T3, total triiodothyronine; T4, total thyroxine; TRH, thyrotropin-releasing hormone; TSH, thyroid-stimulating hormone.

Intravenous administration of nanocurcumin partially reversed dysfunction in the hypothalamic-pituitary-thyroid (HPT) and hypothalamic-pituitary-adrenal (HPA) axes induced by EHS in rats. Compared with in the control group, rats in the EHS group exhibited HPT axis activation accompanied by peripheral hormone conversion abnormalities. Specifically, serum levels of TRH, TSH, T4 and FT4 were significantly increased, whereas T3 and FT3 levels were significantly decreased, consistent with low T3 syndrome (Fig. 4A-F). Physiological saline partially attenuated the alterations in TRH, TSH, T3, T4, and FT4 levels induced by EHS, while FT3 remained unchanged. Nanocurcumin treatment partially corrected these abnormalities, Compared with the EHS group, low-dose nanocurcumin significantly reduced TRH and TSH levels, low- and medium-dose nanocurcumin reduced FT4 levels, medium- and high-dose nanocurcumin reduced T4 levels and increased T3 levels, and high-dose nanocurcumin increased FT3 levels.

In addition, serum CRH and CORT levels were significantly elevated, whereas ACTH was markedly reduced in rats in the EHS group, reflecting impaired feedback regulation of the HPA axis (Fig. 4G-I). Treatment with nanocurcumin partially ameliorated these EHS-induced changes, Among the three doses, high-dose nanocurcumin showed the most comprehensive improvement in HPA axis-related hormone profiles, particularly by restoring ACTH levels and reducing CRH and CORT levels, although low-dose nanocurcumin appeared to exert a stronger effect on CORT reduction. These findings suggested that nanocurcumin has protective and regulatory effects on neuroendocrine dysfunction associated with EHS.

### Nanocurcumin attenuates systemic inflammation and improves serum biochemical markers of peripheral organ and tissue injury in rats with EHS

To further evaluate the systemic effects of nanocurcumin and to determine whether acute toxicity occurred during the short-term observation period, serum inflammatory cytokines and biochemical markers related to peripheral organ and tissue damage were examined.

**Figure 5.**
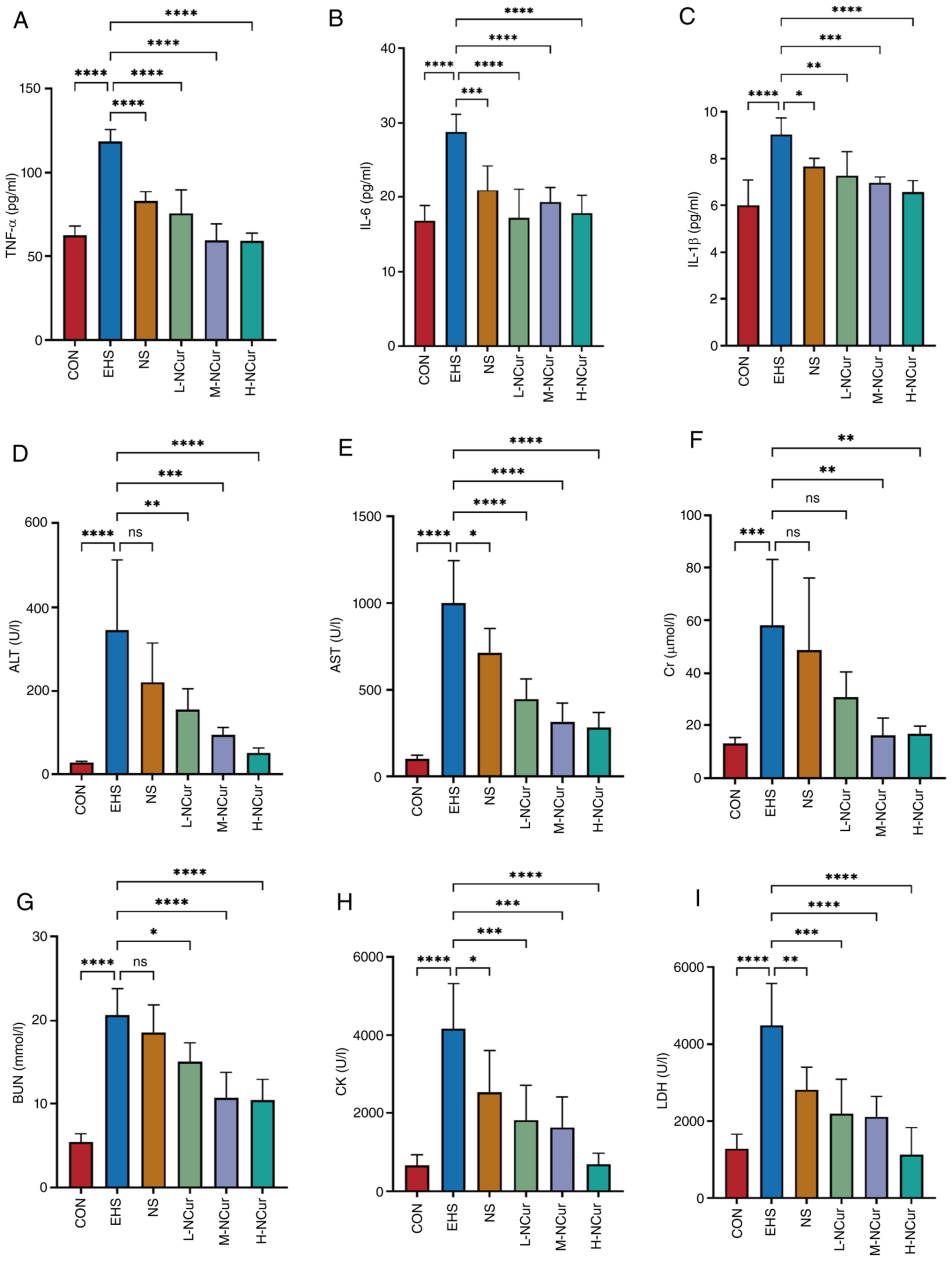
Effect of nanocurcumin on the levels of systemic inflammatory cytokines, and serum biochemical markers related to peripheral organ and tissue injury in rats with EHS. Serum concentrations of (A) TNF-α, (B) IL-6 and (C) IL-1β in different experimental groups. Serum concentrations of (D) ALT, (E) AST, (F) Cr, (G) BUN, (H) CK and (I) LDH in different experimental groups. Data are presented as he mean ± SD. Statistical analyses were performed using one-way ANOVA followed by Tukey's post hoc test. *P<0.05, **P<0.01, ***P<0.001, ****P<0.0001; ns, not significant. ALT, alanine aminotransferase; AST, aspartate aminotransferase; BUN, blood urea nitrogen; CK, creatine kinase; Cr, creatinine; CON, control; EHS, exertional heat stroke; H-NCur, high-dose nanocurcumin; L-NCur, low-dose nanocurcumin; LDH, lactate dehydrogenase; M-NCur, medium-dose nanocurcumin; NS, normal saline.

As shown in Fig. 5A-C, compared with in the control group, rats subjected to EHS exhibited significantly elevated serum levels of TNF-α, IL-6 and IL-1β, indicating a marked systemic inflammatory response. Serum cytokine levels in the NS group showed a partial reduction compared with the EHS group, but did not return to control levels. Conversely, nanocurcumin treatment effectively reduced serum TNF-α, IL-6 and IL-1β levels to varying degrees, with the most pronounced effects observed in the medium- and high-dose groups. These findings suggested that nanocurcumin may attenuate the systemic inflammatory response triggered by EHS.

Additionally, biochemical markers associated with liver, kidney, skeletal muscle and general tissue injury were analyzed. Compared with in the control group, the EHS group demonstrated significant increases in serum ALT, AST, Cr, BUN, CK and LDH levels (Fig. 5D-I). These elevated markers indicated biochemical evidence of liver injury, renal dysfunction, muscle damage and generalized tissue damage following EHS induction. The normal saline group did not exhibit a full reversal of these abnormalities. By contrast, nanocurcumin treatment reduced ALT, AST, BUN, CK and LDH levels in a dose-dependent or partially dose-dependent manner. Serum Cr levels also showed a downward trend following nanocurcumin treatment, especially at medium and high doses. Notably, medium- and high-dose nanocurcumin groups did not exhibit further increases in biochemical markers compared with in the EHS or NS groups. These results indicated that nanocurcumin does not exacerbate acute liver, kidney or tissue injury within the 4-h therapeutic window used in the current study; instead, it partially reverses biochemical abnormalities caused by EHS, demonstrating no apparent acute toxicity.

### Histopathological findings in brain tissue

**Figure 6.**
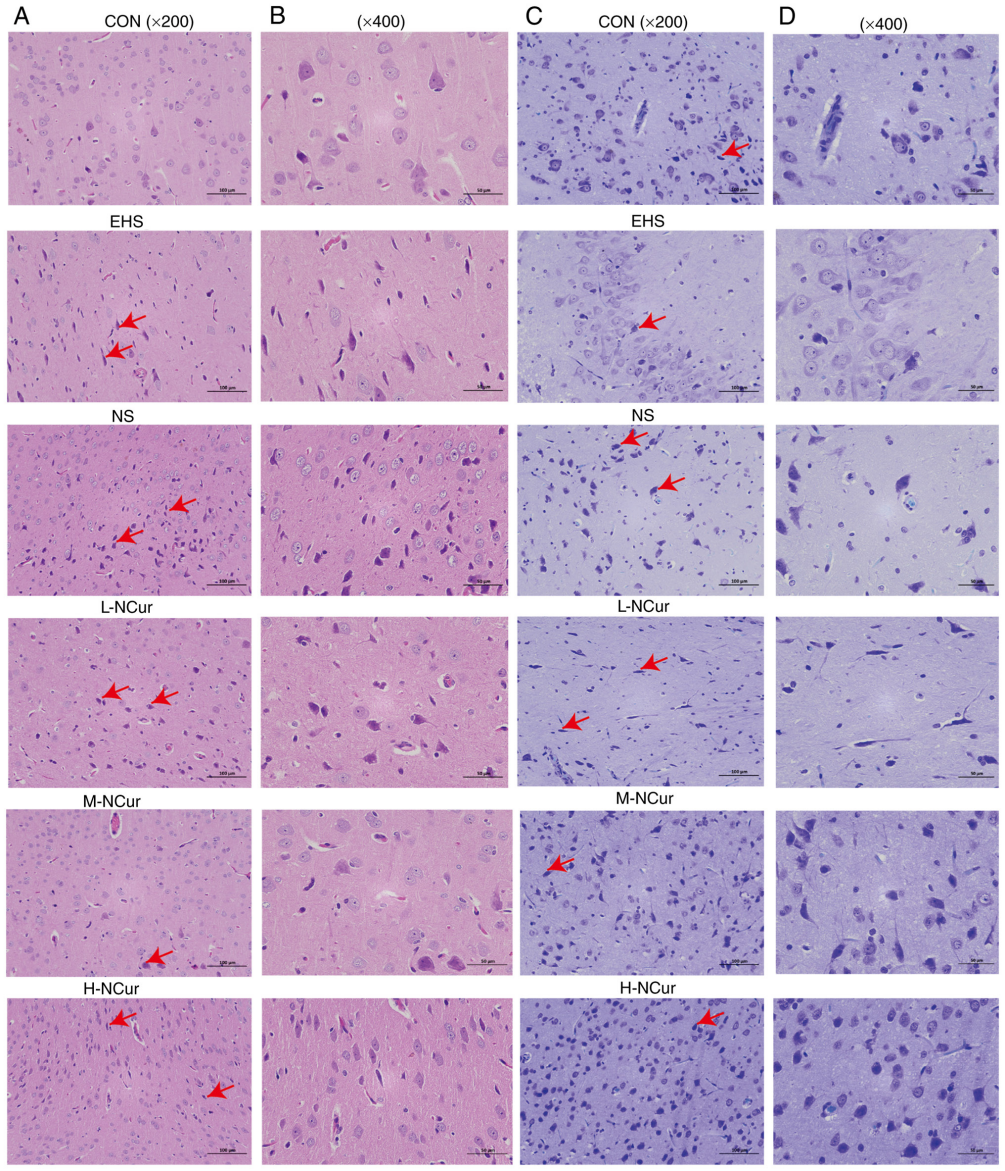
Histopathological analysis of brain tissues using H&E and Nissl staining. H&E staining at (A) ×200 and (B) ×400 magnification. Nissl staining at (C) ×200 and (D) ×400 magnification. Red arrows indicate neuronal shrinkage in both H&E and Nissl staining images. CON, control; EHS, exertional heat stroke; H&E, hematoxylin and eosin; H-NCur, high-dose nanocurcumin; L-NCur, low-dose nanocurcumin; M-NCur, medium-dose nanocurcumin; NS, normal saline.

Histopathological analysis results (H&E staining) are shown in Fig. 6A and B. In the control group, neuronal morphology appeared normal, with intact cellular structures observed in the cerebral cortex and hypothalamus. By contrast, rats in the EHS group exhibited marked neuronal shrinkage, nuclear pyknosis, cytoplasmic eosinophilia and disrupted cellular arrangement, indicating substantial brain damage. Rats in the normal saline group displayed similar pathological changes, indicating no spontaneous recovery. Nanocurcumin treatment attenuated these neuronal injuries in a dose-dependent manner, with the medium- and high-dose groups showing less neuronal shrinkage, improved cellular arrangement and reduced nuclear condensation compared with in the EHS group.

Nissl staining further confirmed these findings (Fig. 6C and D). The EHS group exhibited reduced numbers of Nissl bodies and prominent neuronal atrophy in the hypothalamus. By contrast, nanocurcumin administration maintained neuronal integrity and partially restored Nissl body distribution, especially in the medium- and high-dose groups. These results suggested that nanocurcumin may effectively alleviate EHS-induced neuronal injury in the cerebral cortex and hypothalamus.

### Effect of nanocurcumin on key inflammatory pathway proteins in an EHS model

**Figure 7.**
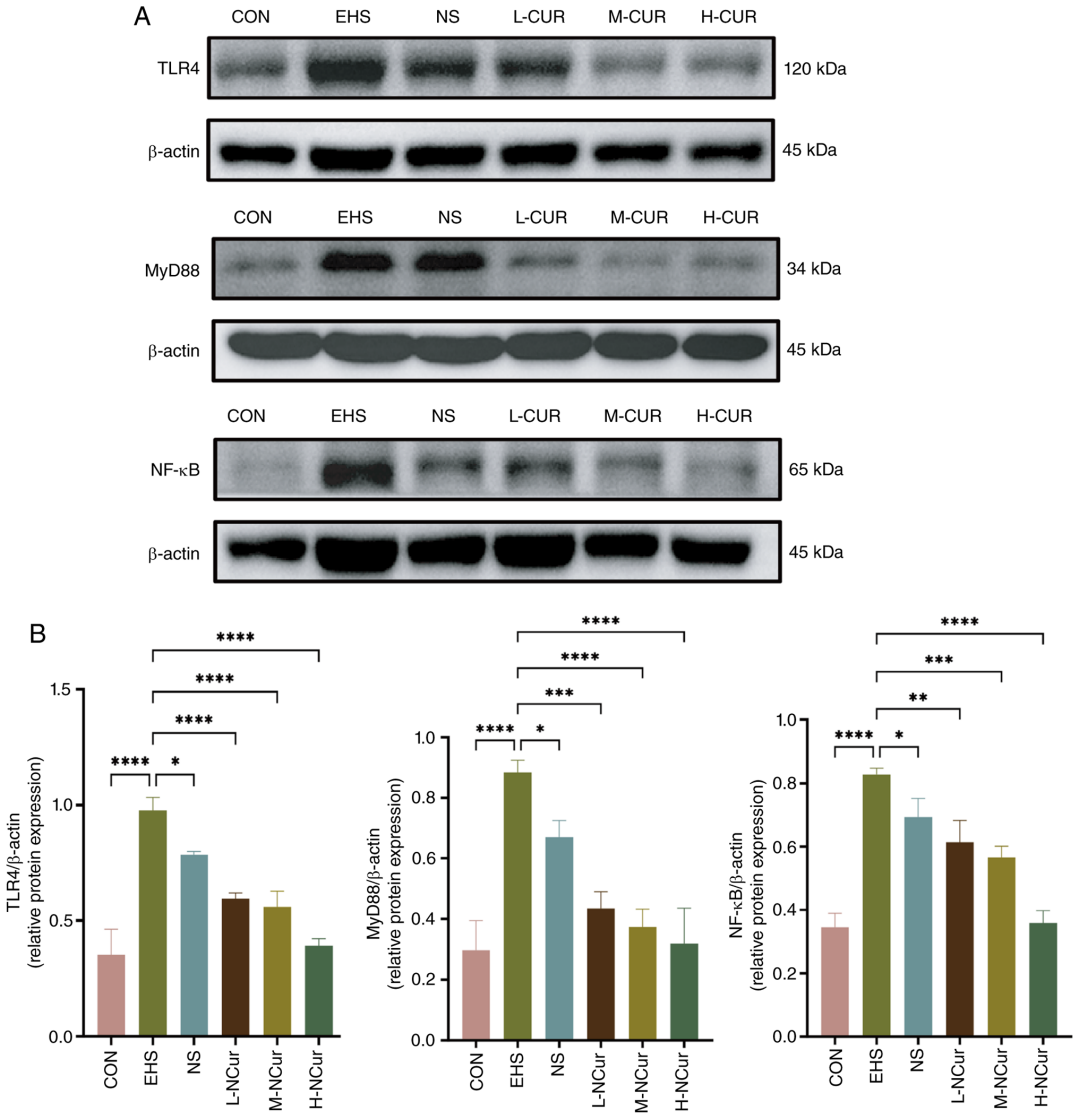
Effect of nanocurcumin on TLR4/MyD88/NF-κB signaling pathway protein expression in the brain tissues of rats with EHS. (A) Representative western blotting images showing MyD88, TLR4, NF-κB and β-actin expression. (B) Semi-quantitative densitometric analysis of MyD88/β-actin, TLR4/β-actin and NF-κB/β-actin ratios. Data are presented as the mean ± SD. Statistical analyses were performed using one-way ANOVA followed by Tukey's post hoc test. *P<0.05, **P<0.01, ***P<0.001, ****P<0.0001. CON, control; EHS, exertional heat stroke; H&E, hematoxylin and eosin; H-NCur, high-dose nanocurcumin; L-NCur, low-dose nanocurcumin; M-NCur, medium-dose nanocurcumin; MyD88, myeloid differentiation factor 88; NS, normal saline; TLR4, Toll-like receptor 4.

To elucidate the therapeutic mechanism of nanocurcumin in EHS, western blot analysis was conducted to measure the expression of key inflammatory signaling proteins in EHS-model rats (Fig. 7). Compared with in the control group, the protein expression levels of TLR4, MyD88 and NF-κB were significantly increased in EHS-model tissues, and normal saline treatment did not restore these parameters to baseline levels. Following nanocurcumin intervention, the expression levels of these proteins decreased in a dose-dependent manner. MyD88, TLR4 and NF-κB expression was significantly decreased in response to all nanocurcumin doses (low, medium and high), with the most pronounced effect observed at the highest dose, nearly restoring expression levels to those of the control group.

## Discussion

EHS is a severe medical emergency characterized by profound systemic effects, particularly severe CNS injury (29,30). The pathophysiological consequences of EHS include systemic inflammatory response syndrome, multi-organ dysfunction syndrome and substantial neuroendocrine dysregulation (31). The CNS is particularly vulnerable to environmental stressors such as hyperthermia, which can disrupt neuroendocrine homeostasis, induce oxidative stress and trigger neuroinflammation (32). The present study provides mechanistic insights into the therapeutic potential of nanocurcumin in EHS, demonstrating its capacity to modulate inflammatory cascades and improve neuroendocrine dysfunction, primarily through inhibition of the TLR4/MyD88/NF-κB signaling axis.

For the present study an integrated approach combining network pharmacology, molecular docking and *in vivo* validation was adopted to elucidate the therapeutic efficacy of nanocurcumin against EHS. Network pharmacology identified key molecular targets involved in EHS pathogenesis, including AKT1, TNF, EGFR, BCL2, STAT3, SRC and NFKB1. Notably, molecular docking analysis demonstrated that curcumin exhibited strong binding affinity for NFKB1, suggesting direct inhibition of the NF-κB pathway, a critical mediator of inflammation in EHS. These findings align with established evidence highlighting the central role of NF-κB in stress-induced inflammatory responses and its involvement in heatstroke pathology (28). By attenuating aberrant NF-κB activation, curcumin potentially reduces systemic inflammation, minimizes tissue damage and facilitates organ recovery.

Molecular docking analyses further supported these findings by demonstrating favorable binding interactions between curcumin and several key EHS-related proteins, including TLR4 and MyD88. These results suggested that curcumin may interact directly with these proteins and thereby influence critical signaling pathways, such as the TLR and NF-κB pathways. Consistent with these observations, KEGG pathway enrichment analysis revealed significant enrichment of inflammation- and immune-related signaling pathways among curcumin targets. This finding is particularly relevant because excessive activation of these pathways is a key contributor to the pathogenesis of EHS, promoting systemic inflammation and subsequent multi-organ injury.

*In vivo* experiments further provided evidence for the neuroprotective effects of nanocurcumin in EHS-induced brain injury. Histopathological analyses demonstrated substantial neuronal damage in the cerebral cortex and hypothalamus following EHS, characterized by neuronal atrophy, cellular shrinkage and disruption of normal cellular architecture. These pathological changes are consistent with typical features of neuronal injury under severe stress conditions (29,33). Notably, nanocurcumin treatment markedly attenuated these alterations, particularly in the hypothalamus, a central regulator of thermoregulation, autonomic function and neuroendocrine activity (34). This observation is of particular importance because hypothalamic dysfunction during EHS may contribute to endocrine disturbances and aggravate systemic injury. The protective effects of nanocurcumin on hypothalamic structure suggest a potential role in preserving hypothalamic function during heat stress and facilitating recovery from EHS.

The endocrine abnormalities observed in rats with EHS, including elevated levels of CRH, CORT, TRH and TSH, together with reduced levels of ACTH, T3 and FT3, indicated substantial dysregulation of the HPA and HPT axes (35). Nanocurcumin treatment partially reversed these hormonal disturbances, suggesting an improvement in neuroendocrine function and adaptive responses to heat stress. This effect may be associated with its protective action on the hypothalamus, as supported by Nissl staining, which demonstrated reduced neuronal atrophy and preservation of neuronal morphology following nanocurcumin administration.

Further evidence for the anti-inflammatory effects of nanocurcumin was provided by the significant suppression of TLR4, MyD88 and NF-κB protein expression in rats with EHS. These findings indicated effective inhibition of the TLR4/MyD88/NF-κB signaling pathway. Because excessive activation of this pathway promotes the production of pro-inflammatory cytokines and contributes to tissue injury and systemic inflammation during EHS, inhibition of this signaling cascade by nanocurcumin may help reduce inflammatory damage and support recovery.

In addition to suppressing inflammatory signaling in brain tissues, the present study demonstrated that nanocurcumin attenuated systemic inflammation and improved serum biochemical markers associated with peripheral organ and tissue injury in rats with EHS. Serum levels of TNF-α, IL-6 and IL-1β increased significantly following EHS induction, reflecting a robust systemic inflammatory response. Nanocurcumin treatment reduced these pro-inflammatory cytokines to varying degrees, indicating its regulatory actions extended beyond the CNS to modulate broader systemic inflammation. Furthermore, rats with EHS exhibited marked elevations in ALT, AST, Cr, BUN, CK and LDH levels, indicative of biochemical abnormalities associated with liver injury, renal dysfunction, skeletal muscle damage and generalized tissue injury. Notably, nanocurcumin administration did not further aggravate these biochemical abnormalities compared with the EHS or normal saline groups. Instead, several parameters showed partial improvement, particularly in the medium- and high-dose groups. These findings suggested that nanocurcumin did not induce additional acute liver, kidney or tissue injury within the 4-h therapeutic window used in the current study, but rather partially reversed biochemical disturbances linked to EHS-induced peripheral organ and tissue damage.

Collectively, network pharmacology analysis and experimental validation highlight the multi-target therapeutic potential of nanocurcumin in EHS. By concurrently modulating key pathways involved in inflammation, oxidative stress and neuroendocrine homeostasis, nanocurcumin represents a promising adjunctive therapy for EHS management. Its dual ability to inhibit the TLR4/MyD88/NF-κB signaling axis and provide neuroprotection against hypothalamic injury provides strong mechanistic rationale for its potential clinical application in mitigating EHS-associated complications.

Despite providing important evidence supporting the protective effects of nanocurcumin against desert dry-heat-induced EHS, the present study has several limitations. First, the post-treatment observation period was restricted to 4 h; thus, the findings primarily reflect early therapeutic efficacy rather than long-term safety or effectiveness. Although serum ALT, AST, Cr, BUN, CK and LDH levels suggested no acute toxicity from nanocurcumin during this brief observation window, formal toxicological evaluations were not conducted. Further studies, including longer observation periods, repeated-dose administration, survival analyses, hematological assessments, behavioral safety evaluations and comprehensive histopathological examinations of peripheral organs, are necessary to fully characterize the safety profile of nanocurcumin. Second, the present study exclusively employed a desert dry-heat-induced EHS model due to its relevance to scenarios involving strenuous physical activity in high-temperature, low-humidity environments, such as military training and outdoor labor in arid environments. Classic or non-EHS models were not included, as establishing these would require distinct passive-heating protocols, additional control and intervention groups, and separate validation of disease severity. Consequently, these findings are specific to desert dry-heat-induced EHS. Whether nanocurcumin exhibits similar protective effects in classic or non-EHS remains uncertain and requires further validation in appropriate comparative models. Third, although serum inflammatory cytokines and biochemical indicators were measured to assess systemic inflammation and peripheral organ injury, extra-cerebral protection was mainly evaluated using circulating biomarkers. Comprehensive tissue-specific pathological and molecular analyses of peripheral organs, including liver, kidney, skeletal muscle, intestine and vascular endothelium, were not performed. Future studies should further clarify the protective effects of nanocurcumin against multi-organ injury using detailed tissue-specific assessments. Finally, while network pharmacology, molecular docking and western blot analyses collectively suggested involvement of the TLR4/MyD88/NF-κB pathway, direct causality was not established through pathway-specific inhibition, gene knockdown or genetic knockout experiments. Thus, future research should include targeted experimental approaches to validate these findings. Further investigations should also explore potential synergistic effects between nanocurcumin and established EHS therapies, and evaluate optimal dosing regimens and translational feasibility.

In conclusion, the present study provides evidence supporting the multi-target therapeutic efficacy of nanocurcumin in EHS, mediated at least partly through suppression of the TLR4/MyD88/NF-κB signaling cascade. By ameliorating neuroendocrine dysregulation, reducing CNS injury and attenuating systemic inflammation, nanocurcumin represents a promising candidate for EHS intervention. These findings establish a scientific basis for advancing nanocurcumin toward clinical application in EHS management and underscore the need for further research to evaluate its combinatorial use with conventional treatment strategies. However, whether these protective effects can be extended to classic or non-EHS requires further validation in appropriate comparative models.

## Figures and Tables

**Table I.** MCODE cluster characteristics of the protein-protein interaction network.

Cluster	MCODE score	Nodes	Edges	Node IDs
1	19.143	22	402	BRAF, CDC42, FOS, EP300, PTGS2, STAT3, TNF, APP, RAF1, NFE2L2, SRC, EGFR, IGF1, HSP90AB1, AKT1, JAK2, GSK3B, IL2, BCL2, RPS6KB1, CHUK, NFKB1
2	6.286	8	44	PSENEN, ADAM10, NCSTN, CTSB, PSEN2, APH1A, MMP14, CDK5
3	6.000	22	126	HSPA1B, MMP9, MMP7, ADAM17, CXCR2, IKBKG, PSEN1, CDK2, CCNA2, MMP13, BACE1, JAK1, NOS2, NOX4, TLR9, AGTR1, SELE, MMP3, IKBKB, SERPINE1, XIAP, CSF1R
4	4.000	4	12	PTGES, PTGS1, ALOX15, PLA2G2A
5	3.000	3	6	DTYMK, DUT, DCK
6	3.000	3	6	ALPL, BST1, ALPI

## Data Availability

The data generated in the present study may be requested from the corresponding author.
